# Loss of PI3K p110*α* in the Adipose Tissue Results in Infertility and Delayed Puberty Onset in Male Mice

**DOI:** 10.1155/2017/3756089

**Published:** 2017-03-05

**Authors:** Victoria L. Boughton Nelson, Ariel L. Negrón, Inefta Reid, Justin A. Thomas, Leon Yang, Richard Z. Lin, Maricedes Acosta-Martínez

**Affiliations:** ^1^Department of Physiology and Biophysics, Stony Brook University Medical Center, Stony Brook, NY, USA; ^2^Graduate Program in Neuroscience, State University of New York at Stony Brook, Stony Brook, NY, USA; ^3^Department of Veterans Affairs Medical Center, Northport, NY, USA

## Abstract

Deletion of PI3K catalytic subunit p110*α* in adipose tissue (aP2-Cre/p110*α*^flx/flx^, *α*−/− hereafter) results in increased adiposity, glucose intolerance, and liver steatosis. Because this endocrine organ releases hormones like leptin, which are important in reproductive physiology, we investigated the reproductive phenotype of *α*−/− males. Compared to controls, *α*−/− males displayed delayed onset of puberty accompanied by a reduction in plasma LH levels and testicular weight. At postnatal day 30, *α*−/− mice exhibited normal body weight but elevated fasted plasma leptin levels. Testicular leptin gene expression was increased, whereas expression of the cholesterol transporter StAR and of P450 cholesterol side chain cleavage enzyme was decreased. Adult *α*−/− males were infertile and exhibited hyperandrogenemia with normal basal LH, FSH, and estradiol levels. However, neither sperm counts nor sperm motility was different between genotypes. The mRNA levels of leptin and of 17-beta-dehydrogenase 3, and enzyme important for testosterone production, were significantly higher in the testis of adult *α*−/− males. The mRNA levels of ER*α*, an important regulator of intratesticular steroidogenesis, were lower in the testis of adult and peripubertal *α*−/− males. We propose that chronic hyperleptinemia contributes to the negative impact that disrupting PI3K signaling in adipocytes has on puberty onset, steroidogenesis, and fertility in males.

## 1. Introduction

The adipose tissue is no longer regarded as a mere reservoir of excess calories in the form of lipids but is now recognized as a very active endocrine organ. Due to its ability to synthesize and secrete a host of endocrine factors called adipokines, the adipose tissue can modulate many physiological processes such as appetite, metabolism, cardiovascular function, and immunity [1]. This ability to communicate with the rest of the body to coordinate nutrition and hormonal signals has made the adipose tissue, particularly the white adipose tissue (WAT), an attractive target to develop novel therapeutics, including adipokine-based strategies, to treat obesity comorbidities like metabolic disorders [1]. However, an often-overlooked function of this endocrine organ is its ability to modulate mammalian reproduction through the cross talk between adipose-derived factors such as leptin and the hypothalamic pituitary-gonadal (HPG) axis [2–4]. Not surprisingly, diseases that result in adipocyte dysfunction, such as obesity, are also accompanied by pubertal and fertility impairment in both sexes [4–8].

The effects of adipokines such as leptin, adiponectin, and resistin on the reproductive axis are mediated through the activation of adipokine receptors that are expressed throughout the HPG axis [2, 3, 9]. The signaling generated by these and other adipokines can modulate gonadotropin release by acting centrally in the hypothalamus or can directly regulate sex steroid production by acting peripherally in the gonads [2, 3, 9]. For example, under normal physiological conditions, leptin signals the HPG axis to indicate the amount of energy stored, serving as a permissive factor for pubertal onset and contributing to the maintenance of fertility in adulthood [4, 9]. On the other hand, conditions of high adiposity like obesity compromise the architecture and function of WAT, resulting in adipocyte hypertrophy, fibrosis, hypoxia, and a deregulated adipokine milieu [1]. Obese individuals, for example, exhibit hyperleptinemia and leptin resistance, which in turn contributes to deficits in pubertal development and adult fertility [5, 8, 10]. Obesity-induced adipocyte hypertrophy is also accompanied by a shift to an adverse adipokine secretory profile that includes elevated levels of proinflammatory factors, such as tumor necrosis factor alpha (TNF*α*), interleukin- (IL-) 1*β*, IL-6, and monocyte chemoattractant protein 1 (MCP1), concomitant with a reduction of anti-inflammatory factors such as IL-10 and adiponectin [1]. The resulting systemic inflammation is thought to contribute to the detrimental effects of obesity on the HPG axis [3]. Furthermore, in addition to its endocrine function, adipose tissue can aromatize androgens to estrogens [2, 11]. Hence, excessive accumulation of fat tissue can result in the pathological accumulation of estrogens that in turn negatively affect gonadotropin release and gonad function. In fact, men suffering from morbid obesity often exhibit reproductive abnormalities such as hypogonadism, hyperestrogenemia, and subfertility associated with lower testosterone (T) production [8, 10–12]. Such a phenotype may be attributed to changes in the synthesis and secretion of different adipose-derived hormones and their modulations of testicular function. Therefore, the adipose tissue is an extragonadal organ that can integrate energy status to reproductive function. Identifying adipose tissue-specific molecular mechanisms making this integration possible will lead to novel approaches that treat obesity related comorbidities including reproductive disorders.

The phosphoinositide 3-kinase (PI3K) signaling cascade is well known for its role in mediating insulin and leptin effects on cell and tissue metabolism [13]. This lipid enzyme is activated in classical metabolic tissues such as the liver, pancreas, skeletal muscle, and adipose tissue. In the latter, PI3K mediates insulin effects on glucose uptake and lipolysis [13–15]. PI3K signaling also participates in the regulation of reproductive function; at the level of the hypothalamus, PI3K regulates the expression of neuropeptides important for fertility and gonadotropin release [16, 17]; at the gonad level, PI3K signaling has been shown to regulate gametogenesis and spermatogenesis [18, 19]. Class 1A PI3Ks are comprised of a catalytic subunit (p110*α*, p110*β*, or p110*δ*) bound to one of several regulatory subunits (collectively referred to as p85) [5]. Pharmacological studies using selective PI3K inhibitors as well as global genetic KO models suggest that p110*α* is the primary insulin-responsive PI3K isoform in adipose tissue [13, 14]. Moreover, our group recently demonstrated the contribution of adipose tissue specific p110*α* to systemic glucose and lipid homeostasis [15]. We showed that mice lacking p110*α* in adipose tissue (*α*−/−) exhibit increased adiposity, glucose intolerance, and liver steatosis [15]. The compromised metabolic phenotype observed in *α*−/− mice was the result of low energy expenditure without changes in food intake or activity [15]. However, in the course of our studies we observed that, in contrast to *α*−/− females, the use of *α*−/− males did not produce litters. Therefore, the goal of the present study is to characterize the reproductive phenotype of *α*−/− male mice. We found that adipose tissue-specific loss of p110*α* results in delayed pubertal onset and infertility in male mice [20]. While not overweight, peripubertal *α*−/− males showed hyperleptinemia and high testicular mRNA expression of leptin. Delayed reproductive maturation in *α*−/− animals was accompanied by low luteinizing hormone (LH) levels and by a reduction in the testicular gene expression of the steroidogenic acute regulatory protein, StAR, and of cytochrome P450 cholesterol side chain cleavage enzyme. Intriguingly and in contrast to models of high-fat diet (HFD) induced obesity, which are usually characterized by low T levels, the infertility observed in *α*−/− males was accompanied by hyperandrogenemia. These findings suggest that disruption of PI3K signaling in adipose tissue interferes with the hormonal feedback pathways that regulate testicular steroid production. Furthermore, our model places adipose tissue-specific PI3K signaling as an integrator of metabolism and reproductive function in males.

## 2. Materials and Methods

### 2.1. Animals

Animals were housed at Stony Brook University, Division of Laboratory Animal Resources, under a 12:12 h light-dark cycle, and had access to water and rodent chow ad libitum. All procedures were approved by the Institutional Animal Care and Use Committee at Stony Brook University Medical Center in accordance with the National Institutes of Health (NIH) Guide for the Care and Use of Laboratory Animals.

p110*α*^flox/flox^ [15] were crossed with aP2-Cre transgenic mice obtained from Bruce Spiegelman [15, 21] to produce experimental aP2-Cre/p110*α*^flox/flox^ and p110*α*^flox/flox^ animals (called *α*−/− and *α*+/+, resp.). These mouse strains were maintained on a mixed genetic background of 129 and C57BL/6 and the control animals for each study were littermates. Animals were screened for the presence of Cre and floxed p110*α* by PCR of isolated genomic tail DNA as described previously [15].

### 2.2. Puberty Assessment and Breeding Studies

Balanopreputial separation was checked daily from weaning by manually retracting the prepuce with gentle pressure. In addition, we assessed the longitudinal change in anogenital distance (AGD).

To test for fertility, at the age of 3 months, *α*+/+ and *α*−/− males were paired with a proven fertile WT female for 7 days, and the female was checked for plugs daily. After 7 days, females were removed and housed separately until a litter was born. Gestation time and litter size were recorded. Wet testicular weights were determined in freshly dissected animals.

### 2.3. Gonad Histology

Testes were fixed in Bouin's reagent. Tissue was embedded in paraffin and cut into 5-*μ*m sections (Histowiz, Brooklyn, NY). Gonads were stained with hematoxylin and eosin [17]. Sections were examined for gross morphological effects.

### 2.4. Sperm Motility and Total Sperm Count

Sperm motility and total count were done as described [22]. Briefly, after removing fat pads surrounding the tissues, epididymides were collected in 1 ml of M2 media (Sigma-Aldrich), at room temperature. One epididymis was cut in half and sperm were expelled by manual pressing. The numbers of motile and immotile sperm were counted in a hemocytometer 15 minutes after sperm were expelled. To immobilize motile sperm for a total sperm count, the hemocytometer was placed for 5 minutes on a 55°C heat block. The second epididymis was chopped into small pieces and left 30 minutes at room temperature. The solution was filtered through a 70 *μ*M filter (Falcon) and the sperm were diluted in PBS before counting the total number of sperm heads in one epididymis. Counting of sperm cells was performed under 40x objective and the concentration and motility of sperms were calculated as million per milliliter (M/ml) and percentage, respectively.

### 2.5. Hormone Assays

Plasma luteinizing hormone (LH) and follicle stimulating hormone (FSH) levels were measured using a Milliplex MAP immunoassay (mouse panel; Millipore) in a Luminex 200 (Luminex Corp; Austin, TX, USA). Plasma testosterone (T) levels were measured by ELISA (R&D Systems, Minneapolis, MN), sensitivity of 0.041 ng/mL. Plasma estradiol (E_2_) was measured by ELISA (Calbiotech, Spring Valley, CA), with sensitivity of <3 pg/ml. Plasma leptin was measured with an ELISA kit (R&D Systems, Minneapolis, MN).

### 2.6. Western Blotting

Testis samples were homogenized in RIPA buffer. After centrifugation, aliquots of supernatant containing equal amounts of protein were subjected to SDS-PAGE and immunoblotting as previously described [15, 17]. p110*α* was detected with a rabbit polyclonal antibody (4255; Cell Signaling). The same blot was hybridized with anti *β*-actin antibody as an internal control (4970; Cell Signaling). Signals were visualized using horseradish peroxidase-linked secondary antibodies and chemiluminescence reagents. Densitometry was performed using Image J (NIH) after imaging with film.

### 2.7. Gene Expression Analysis

Total RNA was extracted using Trizol (Invitrogen), according to the manufacturer's instructions. RNA was isolated from WAT using the RNeasy Lipid Tissue Kit (Qiagen). RNA (2 *μ*g) was reverse transcribed to cDNA using the High Capacity cDNA Transcription kit (Applied Biosystems). Real-Time PCR was performed using a StepOne Plus Real-Time PCR system using PowerUp SYBR Green based gene expression analysis (Life Technologies). Relative gene expression was obtained using the 2^−(ΔΔCT)^ calculation. Samples prepared without reverse transcriptase served as negative controls. *β*-Actin was used as reference gene in all reactions. The primer sequences are listed in Table 1.

### 2.8. Statistics

Results are reported as means ± SEM. Significance was determined by Student's *t*-test, one-way ANOVA, or Mann–Whitney Rank Sum (GraphPad software 5, La Jolla, CA). Statistical significance was accepted when *P* < 0.05.

## 3. Results

### 3.1. Loss of Adipose-Specific p110*α* Results in Delayed Puberty Onset in Males

Adult males with a fat-specific deletion of p110*α* exhibit several metabolic phenotypes characteristic of the metabolic syndrome such as increased body weight and hyperleptinemia [15]. Therefore, we investigated whether these metabolic parameters were affected in *α*−/− peripubertal males. At postnatal day (PND) 30, body weight was similar between *α*+/+ and *α*−/− males (Table 2). A significant increase in fasted plasma leptin levels was observed in *α*−/− compared to *α*+/+ littermates (Table 2). Furthermore, compared to *α*+/+, leptin mRNA expression in WAT of PND 30 *α*−/− was significantly higher, whereas WAT mRNA levels of tumor necrosis factor- (TNF-) *α*, an inflammatory cytokine, were not different between genotypes (Table 2).

**Figure 1:**
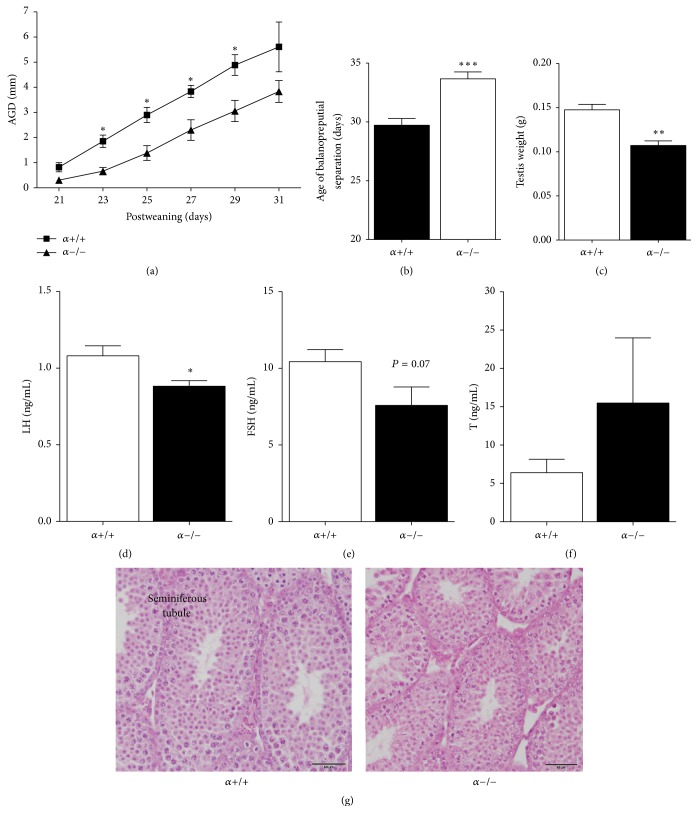
Delayed puberty onset in male *α*−/− mice (aP2-Cre/p110*α*^flx/flx^ are called *α*−/− and control p110*α*^flx/flx^ mice are called *α*+/+). (a) Mean anogenital distance (AGD, mm) measured at postnatal days (PND) 23, 25, 27, 29, 31, and 33. (b) Balanopreputial separation age (*α*+/+, *n* = 14; *α*−/−, *n* = 9). (c) Testis weight (*α*+/+, *n* = 4; *α*−/−, *n* = 5). Plasma LH (d) and FSH (e) levels on PND30 (*n* = 8 per group). (f) Plasma T levels on PND30 (*α*+/+, *n* = 4; *α*−/−, *n* = 5). (g) Representative sections of PND30 testis in *α*+/+ and *α*−/−. Scale bar corresponds to 50 *μ*m. Values are mean ± SEM (Student's *t*-test as compared to control). ^*∗*^*P* < 0.05, ^*∗∗*^*P* < 0.01, ^*∗∗∗*^*P* < 0.001.

To assess the progression of puberty in *α*−/− males we recorded the longitudinal change in anogenital distance (AGD) and day of balanopreputial separation both indicators of activation of the reproductive axis in males that are correlated with androgen exposure or T dependence [30]. AGD was significantly shorter in *α*−/− at PND 23 and continued to be shorter in *α*−/− males than in control males until PND31 (Figure 1(a)). Compared to WT littermates, *α*−/− mice showed a later onset of balanopreputial separation, occurring approximately 3 days later in *α*−/− mice than in control littermates (PND 33.7 ± 0.6 versus PND 29.71 ± 0.6, *P* < 0.001; Figure 1(b)). Testicular weight increases at the time of puberty in response to LH stimulation; therefore we also measured testicular weight at PND30. *α*−/− mice had lower testicular weight compared at PND30 to *α*+/+ controls (Figure 1(c)). Accordingly, plasma LH levels were significantly lower in *α*−/− compared to *α*+/+ at PND30 (Figure 1(d)). On the other hand, FSH levels at PND30 tended to be lower in *α*−/− compared to *α*+/+ males, but it did not reach statistical significance (*α*+/+, 10.4 ± 0.8 ng/mL, *n* = 8 versus *α*−/−, 7.6 ± 1.2 ng/mL, *n* = 8) (Figure 1(e)). In contrast, no genotype effect was observed on circulating T levels at PND30 (Figure 1(f)). In light of their lower testicular weight we sought to investigate whether *α*−/− had abnormal testicular morphology. However, histological evaluation of PND30 testis did not reveal a genotype effect on gross testicular morphology (Figure 1(g)).

### 3.2. Loss of Adipose-Specific p110*α* Results in Infertility and Hyperandrogenemia in Adult Males

In contrast to peripubertal *α*−/− males, adult *α*−/− males showed a significant increase in body weight (Table 2). Fasted circulating leptin levels were significantly higher when compared to those in fasted *α*+/+ males (Table 2). Furthermore, adult *α*−/− males showed increased TNF-*α* gene expression in white adipose tissue (Table 1), consistent with the increased levels of adiposity observed in these mice [15]. Because in humans as well as in rodents, obesity is associated with an increase in circulating estrogen levels [11, 31, 32], we measured circulating levels of E_2_ in adult males. However, there was no difference in plasma E_2_ levels between male *α*−/− and *α*+/+ littermates (Table 2).

**Figure 2:**
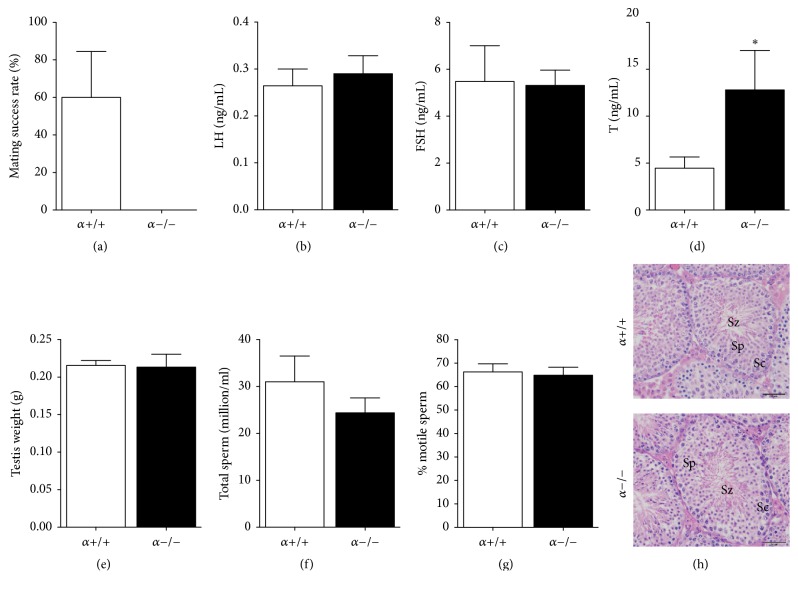
Infertility accompanied by high plasma T levels in male *α*−/− mice. (a) Percent of litter-producing matings by males (*α*+/+, *n* = 5; *α*−/−, *n* = 6). Plasma LH (b) and FSH (c) levels (*n* = 7 per group). (d) Plasma T levels (*α*+/+, *n* = 10; *α*−/−, *n* = 7). (e) Testicular weight (*α*+/+, *n* = 5; *α*−/−, *n* = 4). (f) Total sperm count per epididymis (*α*+/+, *n* = 6; *α*−/−,  *n* = 5). (g) Percentage of motile sperm (*α*+/+, *n* = 7; *α*−/−, *n* = 5). (h) Representative transverse sections of *α*+/+ and *α*−/− mice showing normal seminiferous tubules with all types of spermatogenic elements (Sz, spermatozoa,; Sc, spermatocytes; Sp, spermatids). Scale bar corresponds to 50 *μ*m. Values are mean ± SEM (Student's *t*-test as compared to control). ^*∗*^*P* < 0.05.

In contrast to control males, adult *α*−/− males were not able to sire pups after they were mated with a known fertile female for 7 days (Figure 2(a)). LH and FSH levels in adult *α*−/− males were not significantly different from those in *α*+/+ mice (Figures 2(b) and 2(c)). In contrast, plasma T levels of *α*−/− male mice were significantly higher than those in *α*+/+ littermates (Figure 2(d)).

Although testicular weight was not different between the two genotypes (Figure 2(e)), we investigated whether the infertility observed in *α*−/− males was due to other factors such as a decrease in sperm quality. Hence, we prepared the epididymis for either total sperm count or motility. However, no genotype effects were observed on either total sperm count (Figure 2(f)) or sperm motility (Figure 2(g)). In addition, control and *α*−/− testicular tissue showed normal seminiferous tubules with all types of spermatogenic cells and spermatozoa (Figure 2(h)).

### 3.3. Testicular Gene Expression of Markers of Sex Steroid Production Is Affected in Peripubertal and Adult *α*−/− Males

**Figure 3:**
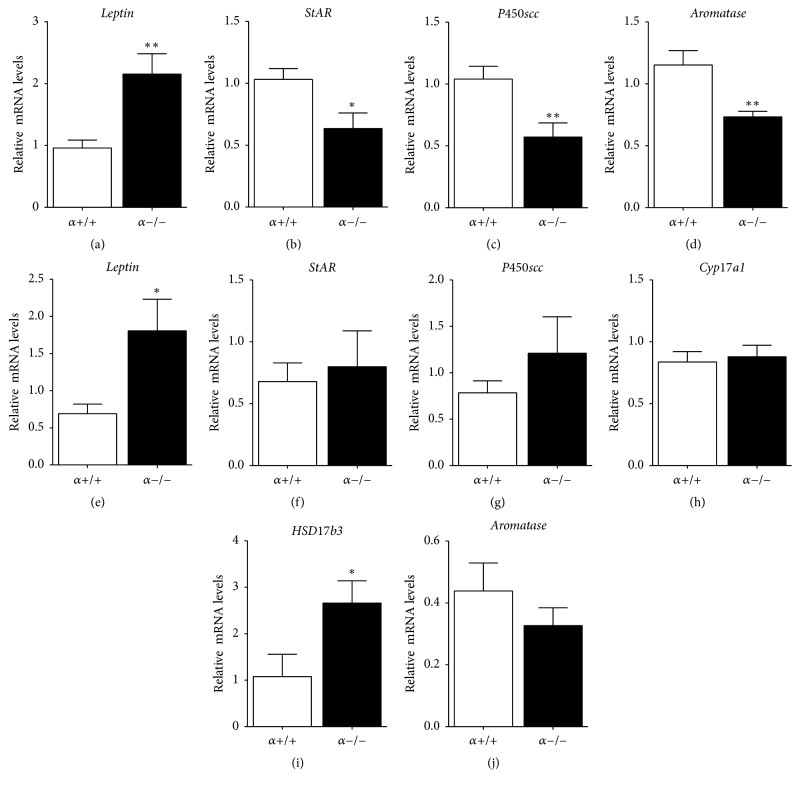
The mRNA expression of leptin, the cholesterol transport protein StAR, and the steroidogenic enzymes P450scc and aromatase normalized to *β*-actin in the testis of peripubertal (PND30) (a–d) and adult (e–g, j) *α*+/+ and *α*−/− males. Leptin mRNA levels in the testis of *α*−/− males were significantly higher in PND30 (a) and adult (e) *α*−/− males. At PND30, testicular mRNA expression of StAR (b), P450scc (c), and aromatase (d) was lower in *α*−/− compared to controls. No genotype effect was observed on the mRNA levels of StAR (f), P450scc (g), or aromatase (j) in adult testes. Gene expression of HSD17b3 (i) but not Cyp17a1 (h) was significantly higher in the testis of adult *α*−/− compared to controls. Values are mean ± SEM (Student's *t*-test as compared to control). ^*∗*^*P* < 0.05, ^*∗∗*^*P* < 0.001; *n* = 10 per genotype from PND30 mice; *n* = 5-6 per genotype from adult animals.

In addition to adipose-derived leptin, testicular leptin regulates T production from Leydig cells through its ability to regulate steroidogenic enzymes [2, 33, 34]. Furthermore, obesity-associated hyperleptinemia negatively impacts testicular function and T production in males [35–37]. Therefore, Real-Time PCR was utilized to investigate whether the hyperleptinemia observed in *α*−/− peripubertal and adult mice was associated with changes in the testicular mRNA expression of leptin and of genes linked to the sequential conversion of steroid precursors to T. Compared to controls, leptin mRNA levels were significantly increased in the testis of both PND30 and adult *α*−/− males (Figures 3(a), and 3(e), resp.). At PND30, testicular mRNA expression of steroidogenic acute regulatory protein (StAR) and of cholesterol side chain cleavage enzyme (P450scc) was significantly decreased in *α*−/− males compared to *α*+/+ controls (Figures 3(b) and 3(c)). Furthermore, the mRNA expression of aromatase, the enzyme responsible for the aromatization of androgens to estrogens, was also significantly decreased in the testis of PND30 *α*−/− males (Figure 3(d)). In contrast, no genotype effects on testicular StAR, P450scc, or aromatase mRNA levels were observed in the testis of adult males (Figures 3(f), 3(g), and 3(j)). However, while no genotype effect was observed in adult testicular expression of cytochrome P450 17A1 (*Cyp17a1*) the mRNA levels of 17-beta-dehydrogenase 3 (*Hsd17b3*) were significantly increased in the testis of *α*−/− compared to *α*+/+ controls (Figures 3(h) and 3(i)).

### 3.4. ER*α* mRNA Levels Are Decreased in the Testis of PND30 and Adult *α*−/− Males

**Figure 4:**
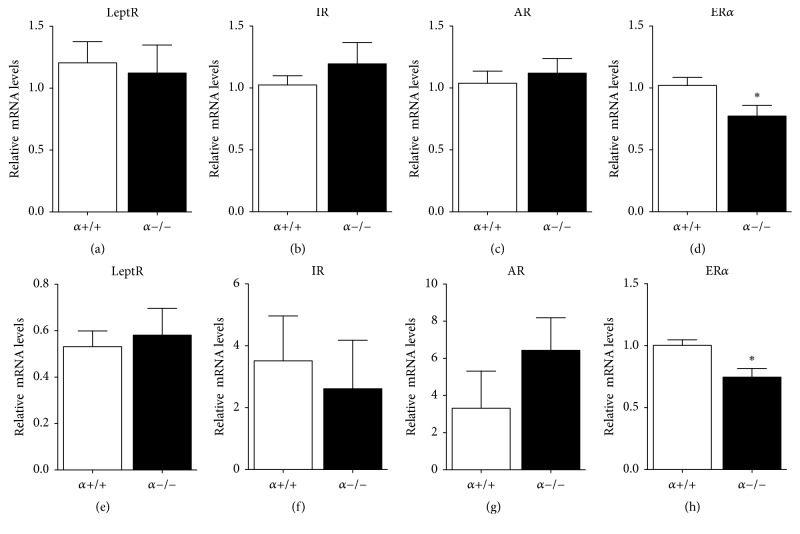
The mRNA levels of ER*α* are lower in the testis of PND30 and adult *α*−/− males compared to controls. Relative mRNA levels of testicular LepR (a), the IR (b), the AR (c), and ER*α* (d) normalized to *β*-actin in PND30 males. Relative mRNA levels of testicular LepR (e), the IR (f), the AR (g), and ER*α* (h) normalized to *β*-actin in adult males. Values are mean ± SEM (Student's *t*-test as compared to control). ^*∗*^*P* < 0.05, *n* = 10 per genotype from PND30 mice; *n* = 5-6 per genotype from adult animals.

Activation of leptin receptor (LepR) signaling is only one of various regulatory pathways that can influence steroidogenic genes and hence production of T by the testis. Therefore, we investigated whether testicular gene expression of metabolic and steroid hormone receptors was affected by deletion of p110*α* in adipose tissue. There was no genotype effect observed on the testis mRNA levels of the leptin, insulin, or androgen receptors at PND30 (Figures 4(a)–4(c)). Similarly, no genotype effect was observed on the expression of these receptors in the testis of adult animals (Figures 4(e)–4(g)). Intriguingly, testicular mRNA levels of estrogen receptor alpha (ER*α*) were decreased in both PND30 and adult males (Figures 4(d) and 4(f), resp.).

### 3.5. Protein Expression of p110*α* Is Not Disrupted in the Testis of *α*−/− Males

**Figure 5:**
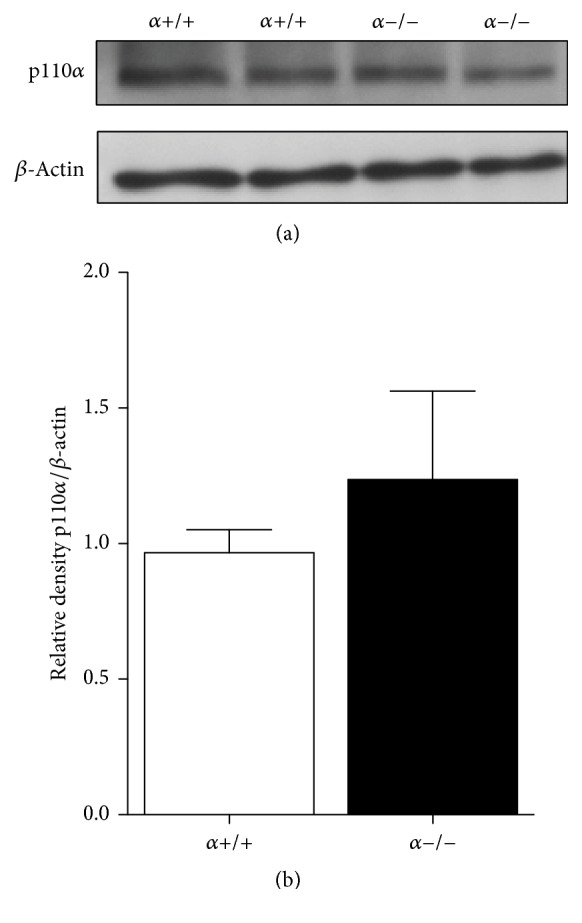
p110*α* protein expression in the testis from PND30 *α*+/+ and *α*−/− mice. (a) Representative immunoblots of p110*α* from protein isolated from the testis of PND30 *α*+/+ and *α*−/− mice. Western blots were quantified (b) by normalizing to *β*-actin loading control (*n* = 4 per group).

A recent study showed that homozygous mice with a kinase-dead knocking allele of p110*α* were embryonic lethal whereas heterozygous males were subfertile with reduced testis size [19]. In published work we used Western blotting to confirm reduced p110*α* protein expression in adipocytes isolated from WAT and BAT of *α*−/− mice compared with *α*+/+ animals with no genotype effects in p110*α* protein expression in liver or muscle [15]. As shown previously we confirmed reduced p110*α* protein expression in adipocytes isolated from knockout WAT using Western blotting (Supplemental Figure 1 in Supplementary Material available online at https://doi.org/10.1155/2017/3756089). Extending these findings we now show via Western blotting that p110*α* protein expression is not different in the testis of *α*+/+ and *α*−/− mice (Figures 5(a) and 5(b)). These results further confirm the specificity of aP2 Cre-mediated p110*α* deletion and that the reproductive phenotypes observed in *α*−/− males are not due to p110*α* deletion in the gonads.

## 4. Discussion

In the present study, we show that deletion of class IA PI3K catalytic subunit p110*α* in adipose tissue results in delayed puberty onset and impaired fertility in males, with adults also showing hyperandrogenemia. The PI3K signaling pathway participates in the regulation of specific aspects of male reproductive function such as gonadotropin release [16, 17], gonadal development [18], and spermatogenesis [19] acting on both the hypothalamic and the gonad level. To our knowledge, this is the first report to demonstrate that adipose tissue-specific disruption of PI3K signaling impairs male reproductive capacity, independent of a diet manipulation.

In mammals, puberty starts with the pulsatile secretion of hypothalamic gonadotropin releasing hormone (GnRH) [9, 38]. While the mechanisms that trigger GnRH secretion are not completely clear, peripherally produced signals such as leptin contribute to the onset and maturation of the HPG axis. Serum leptin levels are positively correlated with the amount of body fat, and prepubertal body composition affects the progression of pubertal development [38–40]. Although no longer thought to be the trigger for pubertal onset, leptin is an important permissive factor. For example, mice with congenital deficiency of leptin (*ob/ob* mice) exhibit hyperphagia, reduced energy expenditure, obesity, and infertility [41]. Administration of leptin to* ob/ob* mice restores their fertility [33, 42, 43], and in normal animals, it can advance puberty onset [44]. On the other hand, deregulated leptin production, especially at an early developmental stage, can have detrimental effects on the maturation and function of the HPG axis [45]. We did not observe a genotype effect on body weight in peripubertal males. However, higher serum leptin levels as well as a higher leptin mRNA expression in the WAT and testis accompanied the pubertal delay, decreased testicular mass, and reduced gonadotropin levels in PND30 *α*−/− animals. It is likely that the proportion of fat mass to lean mass is higher in *α*−/− at this age, as was observed in adult *α*−/− animals [15]. In humans there is an increase in leptin levels in males from prepuberty to early puberty, followed by a decline that coincides with increased T and testicular volume [39, 40]. However, it has been reported that the expected decline of leptin levels occurring with the progression of puberty is not observed in males with delayed puberty [46]. Furthermore, the pubertal phenotype observed in *α*−/− male mice bares resemblance to findings from epidemiological studies linking obesity to a higher risk for pubertal delay in boys [6, 7]. Therefore, our findings suggest that hyperleptinemia contributes to the delay in pubertal onset observed in *α*−/− males.

The effects of leptin are mediated by its specific receptor (LepR) in target tissues including the hypothalamus, pituitary, and the gonads [3, 9]. The actions of leptin on the HPG axis are complex and whether its effects are stimulatory or inhibitory depends on the concentration and duration, as well as the developmental state. For example, in vivo and in vitro studies suggest that at low concentrations leptin induces testicular T secretion, whereas high leptin concentrations are inhibitory [9, 33, 45, 47, 48]. The LepR is expressed in the testis and circulating leptin can cross the blood-testis barrier [49–52]. Furthermore, testicular cells, including Leydig cells, which are responsible for T production, also produce leptin [2, 53]. Compared to controls, PND30 *α*−/− males showed lower testicular mass; however lower free circulating T levels were not observed. This is not surprising, as peripheral circulating T levels do not always coincide with intratesticular levels. Leptin can regulate Leydig cell's steroid production through the transcriptional regulation of different steroidogenic genes such as those encoding the steroid acute regulatory protein (*Star*) and the cholesterol side chain cleavage enzyme P450ssc (encoded by* Cyp11a1* gene). The StAR protein participates in the entry of cholesterol inside the mitochondria, a rate-limiting step of Leydig cell steroid production, whereas P450scc, located in the mitochondria, catalyzes the conversion of cholesterol to pregnenolone. We found that the hyperleptinemia and the high intratesticular leptin gene expression in PND30 *α*−/− males coincided with a significant decrease in testicular mRNA expression of StAR and P450scc. Our findings are in agreement with in vivo and in vitro studies showing that high leptin levels result in lower testicular expression of these and other steroidogenic enzymes [34, 54].

In contrast to PND30 *α*−/− males, adult *α*−/− males exhibited higher body weight and elevated WAT mRNA expression of the inflammatory cytokine TNF-*α*. Together with our previous findings [15], adult *α*−/− males showed a metabolic phenotype that includes characteristics of obesity related metabolic syndrome, such as reduced energy expenditure, insulin resistance, hyperleptinemia, and a proinflammatory state [1]. Here, we report that, in addition to their metabolic phenotype, *α*−/− males are also infertile and exhibit high circulating T levels compared to controls. This was unexpected, as high body weight and hyperleptinemia are inversely correlated with T production in both humans and murine models [8, 10, 12, 31, 32, 55, 56]. The WAT is capable of converting T to estrogen and the pathological accumulation of fat tissue during obesity can result in excessive estrogen production [11, 31, 32]. Estrogen in turn exerts negative feedback on LH and T production. However, higher estrogen levels were not observed in adult *α*−/− males. Based on studies in humans and rodent models of diet-induced obesity, a decrease in T levels is likely to be proportional to the degree of obesity [31, 57]. Even though adult *α*−/− males are heavier than their control littermates ([15] and present study), we think that our model does not represent extreme obesity, and the fat accumulation observed in adult *α*−/− males is not sufficient to produce high estrogen levels. In fact, recent studies in rodents have shown that whether or not overnutrition results in male infertility and in alterations in reproductive parameters, such as higher estrogen levels, lower gonadotropin, and T levels, depends on the developmental period at which the animal has access to excessive caloric intake (before puberty or as an adult) in addition to how long (chronic versus short-term) the access to excessive caloric intake is maintained. For example, male rats exposed to overnutrition postnatally and into adulthood exhibit low LH and FSH levels accompanied by low T and lower testicular expression of steroidogenic enzymes [55]. On the other hand, a more variable reproductive response is observed in male rats exposed to excessive caloric intake in the form of a HFD as adults, with some studies reporting higher E_2_ levels without effects on LH, FSH, or T [31]. Other studies showed reduced gonadotropins and T levels after a HFD [32]. On the other hand, the early exposure to high leptin levels might have contributed to the high T levels observed in adult *α*−/− males. For instance, we found that testicular aromatase mRNA levels were lower in PND30 *α*−/− males compared to controls. Aromatase (encoded by* Cyp19* gene) converts androgens into estrogens and estrogen in turn directly regulates testicular steroid production [58]. In fact, throughout development paracrine and autocrine effects of locally produced estrogens are in part responsible for fine-tuning steroidogenesis and hence T production [58, 59]. It is possible that the changes in testicular steroidogenic enzymes mRNA levels we observed in PND30 *α*−/− males are part of a developmental shift that allows for the increased T production observed in adult *α*−/− males.

The infertility and hyperandrogenemic phenotype observed in adult *α*−/− males is very similar to that observed in male mice with global deletion of the gene encoding estrogen receptor alpha (*Esr1*), the estrogen receptor responsible for mediating the majority of estrogen's effects on the HPG axis [60–62]. Similar to *α*−/− males, ER*α*KO males are overweight, with adipose hyperplasia, and are infertile with high T levels despite normal LH and FSH levels [61, 63]. We hypothesized that the high T levels observed in adult *α*−/− males may be due to changes in ER*α* signaling. We found that the testicular mRNA levels of ER*α* were lower in *α*−/− males compared to controls. This genotype effect was observed in the testis of both PND30 and adult *α*−/− animals. A number of factors could have contributed to a decreased testicular ER*α* gene expression in *α*−/− males including an increase in inflammatory signals [64]. However, ER*α* deficiency is known to enhance androgen biosynthesis in the mouse Leydig cell [65]. Genetic or pharmacological blockade of ER*α* results in higher steroidogenic enzyme activity as well as higher mRNA levels for key steroidogenic enzymes such as cytochrome P450 17*α*-hydroxylase/17-20 lyase (P40_17*α*_) and 17*β*-dehydrogenase type 3 [58, 59, 65]. In our study, among the steroidogenic enzymes examined, only the gene expression of 17*β*-dehydrogenase 3 was significantly higher in the testis of adult *α*−/− mice. This enzyme encoded by the Hsd17b3 gene is predominantly expressed in the testis and catalyzes the conversion of androstenedione to T. Therefore the increase of Hsd17b3 in the testis of adult *α*−/− mice explains high T levels observed. While we did not observe a genotype effect on the mRNA levels of other steroid producing enzymes in adult testis, it is important to point out that an absence of gene expression does not necessarily mean that no effects on protein or enzyme activity are occurring. We suggest that, in the absence of a genotype effect on LH levels, the lower intratesticular expression of ER*α* contributes to the hyperandrogenemia observed in *α*−/− males.

We cannot exclude the possibility that the lower LH levels observed in *α*−/− PND30 males are the result of leptin and/or other adipose-derived factor effects at the pituitary and/or the hypothalamic level. For example, leptin in a dose-dependent manner has been shown to selectively inhibit in vitro gonadotropin secretion by hemipituitaries from adult fasted rats [48]. On the other hand, hypothalamic neurons that play a crucial role in pubertal development such as kisspeptin-expressing neurons are also targets of leptin action [9]. However, differences in hypothalamic* Kiss1* mRNA levels between control and *α*−/− males were not detected (unpublished results). Furthermore, contrary to the effects on the gonads and pituitary, the central actions of leptin are stimulatory on GnRH/LH release, and PND30 *α*−/− males showed lower LH. There is evidence that the *α*−/− mice have central leptin resistance given that leptin levels are elevated without eliciting a change in eating behavior [15]. While we do not see a change in hypothalamic* Kiss1* gene expression the changes in LH and FSH prior to obesity onset point towards impaired central leptin signaling. It is also possible that, in the hypothalamus, leptin-independent factors such as those regulated by estrogen through ER*α* are playing a role in the phenotype observed in *α*−/− males.

The deletion of p110*α* in adipose tissue affects the levels of other adipose-derived peptides such as resistin, whose role in testicular steroid production is not clear [2]. Moreover, we cannot rule out that the effects of adipose tissue specific deletion of p110*α* on male puberty and fertility are the result of the combined effects of multiple adipose-derived hormones, including leptin, adiponectin, and resistin. Additionally, high insulin levels in *α*−/− adult males might be another contributing factor to hyperandrogenemia, as pharmacological doses of insulin are known to stimulate androgen production [66].

Finally, the inability of *α*−/− males to sire pups in proven fertile females could not be explained by impairments in spermatogenesis or sperm motility, as both parameters were similar between genotypes. It is possible that impaired male sexual behavior such as lower mounting and intromission frequency could explain the infertility observed in *α*−/− males. Impairments in sexual behavior have been observed in rodent models of HFD-induced obesity as well as in genetic models of obesity such as the ER*α*KO males [61, 67]. On the other hand, the production of reactive oxygen species in obese models can also impair the quality of the sperm produced, resulting in subfertility [8, 10, 37, 68]. These possibilities will be worth exploring in future studies.

## 5. Conclusion

The present study introduces a novel genetic model linking a specific signaling pathway, PI3K, in the adipose tissue with the modulation of different reproductive parameters in males independent of a dietary manipulation. Our findings support the emerging research stressing the effects of overnutrition prior to puberty and demonstrate the repercussions of errant adipose tissue function that are imprinted during postnatal development and last long into adulthood. This model places adipose tissue-specific PI3K signaling as an integrator of metabolism and reproductive function in males.

## Supplementary Material

White adipose tissue loss of p110*α* protein.

## Figures and Tables

**Table 1:** Primer sequences for the genes tested and references.

Gene	Forward primer	Reverse primer	Reference
StAR (*Star*)	ACAACCAGGAAGGCTGGAAG	ATGCAGGTGGGGCCGTGTTCA	[23]
P450scc protein (*Cyp11a1*)	AGGTCCTTCAATGAGATCCCTT	TCCCTGTAAATGGGGCCATAC	[23]
Aromatase (*Cyp19a1*)	TGTGTTGACCCTCATGAGACA	CTTGACGGATCGTTCATACTTC	[24]
17*α*-Hydroxylase (*Cyp17a1*)	GGAGAGTTTGCCATCCCGAA	CGCTCAGGCATAAACCGAT	[25]
17-beta-hydroxysteroid dehydrogenase (*Hsd17b3*)	ATTTTACCAGAGAAGACATCT	GGGGTCAGCACCTGAATAATG	[23]
Leptin (*Lep*)	TGACACCAAAACCCTCATCA	AGCCCAGGAATGAAGTCCA	[26]
TNF-*α* (*Tnf*)	CCACCACGCTCTTCTGTCTA	AGCTGCTCCTCCACTTGGTG	[26]
LepR (*Lepr*)	AGAACGGACACTCTTTGAAGTCT	AACCATAGTTTAGGTTTGTTTC	[27]
IR (*Insr*)	GAGAGGATGTGAGACGACG	CAGGTTGTTCCGGATGTCC	[26]
AR (*Ar*)	TGCCCGAATGCAAAGGTCTT	TTGGCGTAACCTCCCTTGAAA	[28]
ER*α* (*Esr1*)	GGTGCCCTACTACCTGGAG	GCCCACTTCGTAACACTTGCGC	[29]
*β*-Actin (*Actb*)	AGGCCCAGAGCAAGAGAG	GGGTGTTGAAGGTCTCAAAC	[26]

StAR, steroidogenic acute regulatory enzyme, LepR, leptin receptor, IR, insulin receptor, AR, androgen receptor, ER*α*, estrogen receptor alpha, TNF-*α*, tumor necrosis factor. In parenthesis is the gene encoding the protein.

**Table 2:** Body weight, plasma leptin and estradiol levels, and white adipose tissue mRNA levels of leptin and TNF-*α* in PND30 and adult *α*+/+ and *α*−/− males.

	PND30		Adult (3-4 mo.)	
	*α*+/+	*α*−/−	*α*+/+	*α*−/−
Body weight (g)	16.3 ± 0.7 (*n* = 6)	14.64 ± 1.0 (*n* = 5)	26.27 ± 0.5 (*n* = 7)	30.67 ± 1.4^*∗∗*^ (*n* = 6)
Fasted plasma leptin (ng/mL)	1.03 ± 0.02 (*n* = 4)	1.2 ± 0.05^*∗*^ (*n* = 5)	1.45 ± 0.3 (*n* = 6)	10.30 ± 2.5^*∗∗*^ (*n* = 7)
Plasma E_2_ (pg/mL)	NA	NA	2.1 ± 0.3 (*n* = 6)	2.3 ± 0.3 (*n* = 5)
WAT leptin (a.u) (fasted)	1.3 ± 0.1 (*n* = 3)	20.0 ± 4.8^*∗*^ (*n* = 3)	NA	NA
WAT TNF-*α* (a.u) (nonfasted)	1.2 ± 0.2 (*n* = 9)	0.9 ± 0.1 (*n* = 9)	0.8 ± 0.1 (*n* = 5)	2.1 ± 0.2^*∗∗*^ (*n* = 3)

Data are means ± SEM. aP2-Cre/p110*α*^flox/flox^ mice are referred to as *α*−/−; control p110*α*^flox/flox^ mice are referred to as *α*+/+. ^*∗*^*P* < 0.05 and ^*∗∗*^*P* < 0.01. E_2_, estradiol, WAT, white adipose tissue, NA, not available.
