# Supplementary material for: Loss of PI3K p110α in the Adipose Tissue Results in Infertility and Delayed Puberty Onset in Male Mice
Source: Biomed Res Int. 2017 Mar 5;2017:3756089. doi: 10.1155/2017/3756089 (PMC5357525; doi:10.1155/2017/3756089)
Supplement: Supplementary file 1 — White adipose tissue loss of p110α protein. [file 3756089.f1.zip › New Microsoft Word Document.docx]

**Supplemental Fig. 1:** Adipose tissue-specific loss of p110α protein. Adipocytes were isolated from gonadal white fat pads of adult p110α knockout mice and their control (as described in 15). Isolated adipocytes from several animals of each genotype were pooled and subjected to Western blotting using a p110α specific antibody (Cell Signaling Antibody #4255, 1:1,000). β-actin (Cell Signaling, # 4970) served as a loading control. Relative density normalized to β-actin: 1.3 vs. 0.6 for controls (α+/+) and knockouts (α -/-), respectively.

**Supplemental Methods:**

**Preparation of isolated adipocytes**

WAT was collected from mice anesthetized with ketamine-xylazine. White adipocytes were isolated with 3 mg/ml type I collagenase (Worthington Biomedical) in Krebs-Ringer bicarbonate solution-HEPES buffer, pH 7.4 (supplemented with 3% fatty acid-free bovine serum albumin). Adipocytes were filtered through 297-**μ**m polypropylene mesh, washed three times in Krebs-Ringer bicarbonate-HEPES buffer, and collected for experiments
