# Supplementary figures and images for: Loss of PI3K p110α in the Adipose Tissue Results in Infertility and Delayed Puberty Onset in Male Mice
Source: Biomed Res Int. 2017 Mar 5;2017:3756089. doi: 10.1155/2017/3756089 (PMC5357525; doi:10.1155/2017/3756089)

$\alpha^{+}/+$

$\alpha^{-}/-$

p110 $\alpha$

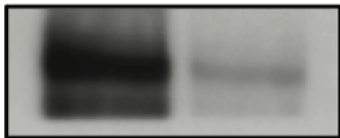

$\beta$ -actin

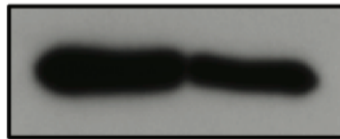

Supplement: Supplementary file 1 — White adipose tissue loss of p110α protein. [file 3756089.f1.zip › WB for Supp Fig 1_BMRI_1859526.pdf]
